# Development and characterization of formulations based on combinatorial potential of antivirals against genital herpes

**DOI:** 10.1007/s00210-024-03468-y

**Published:** 2024-09-30

**Authors:** Mahesh Gaikwad, Amal George, Aparna Sivadas, Kavitha Karunakaran, Sudheesh N, Siddappa N. Byradeddy, Chiranjay Mukhopadhyay, Piya Paul Mudgal, Madhur Kulkarni

**Affiliations:** 1 SCES’s Indira College of Pharmacy, New Mumbai Pune Highway, Tathawade, Pune, India; 2 Manipal Institute of Virology, Manipal Academy of Higher Education, Manipal, India; 3 Department of Pharmacology and Experimental Neuroscience, University of Nebraska Medical Centre, Omaha, NE USA

**Keywords:** Genital herpes, Antivirals, Local drug delivery, Combination therapy, Formulations, Good health and well-being

## Abstract

Herpes simplex virus type 2 (HSV-2) treatment faces challenges due to antiviral resistance and systemic side effects of oral therapies. Local delivery of antiviral agents, such as tenofovir (TDF) and zinc acetate dihydrate (ZAD), may offer improved efficacy and reduced systemic toxicity. This study’s objective is to develop and evaluate local unit dose formulations of TDF and ZAD combination for local treatment of HSV-2 infection and exploring their individual and combinatory effects in vitro. The study involved the development of immediate-release film and pessary formulations containing TDF and ZAD. These formulations were characterized for physicochemical properties and in vitro drug release profiles. Cytotoxicity and antiviral activity assays were conducted to evaluate the individual and combinatory effects of TDF and ZAD. Film formulations released over 90% of the drugs within 1 h, and pessary formulations within 90 min, ensuring effective local drug delivery. ZAD showed moderate antiviral activity while TDF exhibited significant antiviral activity at non-cytotoxic concentrations. The combination of TDF and ZAD demonstrated synergistic effects in co-infection treatments, reducing the concentration required for 50% inhibition of HSV-2. Developed film and pessary formulations offer consistent and predictable local drug delivery, enhancing antiviral efficacy while minimizing systemic side effects. The combination of TDF and ZAD showed potential synergy against HSV-2, particularly in co-infection treatments. Further preclinical studies on pharmacokinetics, safety, and efficacy are necessary to advance these formulations toward clinical application.

**Supplementary Information:**

The online version contains supplementary material available at 10.1007/s00210-024-03468-y.

## Background

The Herpesviridae family is categorized into alpha (α), beta (β), and gamma (γ) herpesviruses based on their genetic organization, replication strategies, and host range. The α-herpesviruses include herpes simplex virus 1 and 2 (HSV-1, HSV-2) and varicella zoster virus (VZV) (Sehrawat et al. 2018). HSV-2 is an enveloped, double-stranded DNA virus that causes herpes genitalis—a globally disseminated infection commonly manifesting as lesions in the anogenital regions of affected individuals (Sauerbrei 2016). HSV-2 is sexually transmitted and capable of establishing latency within peripheral neurons, with the potential for reactivation (Cohen 2020). Several antiviral drugs, including acyclovir, famciclovir, and valaciclovir, are prescribed for treating most HSV-2 cases (Kimberlin and Whitley 2007). However, certain mutations in viral genes, such as UL23 and UL30, which encode viral proteins, can reduce or nullify the efficacy of these antivirals (Giorgi et al. 2023). Although the reported prevalence of drug resistance in the immunocompetent population is low (0.1–0.6%), studies show a significantly higher prevalence (3.5–10%) in immunocompromised groups, such as hematopoietic stem cell transplant recipients, anti-cancer chemotherapy recipients, HIV-positive individuals, and organ transplant recipients. In some cohorts, like allogenic bone marrow transplant patients, resistance prevalence can reach up to 30% (Jiang et al. 2016). This makes antiviral resistance particularly threatening to immunocompromised populations (Gershengorn and Blower 2000).

Long-term oral therapy, essential for the treatment and prophylaxis of HSV-2 infection, is associated with several systemic side effects, such as renal and bone toxicity (Kenzaka et al. 2021; Hammond et al. 2024; Fleischer and Johnson 2010; Stern et al. 2021; Brandariz-Nuñez et al. 2021). While tenofovir disoproxil fumarate (TDF) and zinc acetate dihydrate (ZAD) are FDA-approved for oral treatment, they are not approved for HSV-2 treatment. (Fernández-Romero et al. 2012; Tyo et al. 2017). Achieving therapeutic efficacy with TDF requires strict daily adherence, but compliance can be challenging. Moreover, higher oral doses are needed to reach effective concentrations, particularly in the cervicovaginal region, due to the first-pass effect. To overcome these drawbacks of oral therapy, several attempts have been made to deliver these drugs locally to the cervicovaginal and colorectal regions. For instance, vaginal administration of tenofovir has been found to achieve 1000 times higher concentrations at the site of action compared to oral administration (Robinson et al. 2018). The CAPRISA 004 trial, conducted among South African women at high risk of HIV-1 infection, involved postcoital vaginal administration of 1% tenofovir gel. Women who adhered to the therapy had a 51% lower incidence of HSV-2 infection acquisition (Bender Ignacio et al. 2015). Similarly, the VOICE trial, which involved a larger sample size, indicated a 40% lower acquisition rate among women who regularly used tenofovir gel (Marrazzo et al. 2019). Furthermore, preclinical assessments of a ZAD carrageenan vaginal gel in macaque and mouse models demonstrated significant protection against HSV-2 viral challenge (Fernández-Romero et al. 2012). Phase 1 clinical studies of a carrageenan gel–based formulation containing ZAD and MIV-150 have proven the safety and tolerability of vaginal administration of these drugs (Friedland et al. 1999).

In recent years, combination therapies for viral infections have gained attention for their ability to enhance effectiveness, combat drug resistance, and reduce adverse effects compared to single-drug treatments (Ianevski et al. 2022). These therapies, proven effective against viruses like HIV and HCV, often involve using multiple antiviral drugs simultaneously (Geddawy et al. 2017; Shyr et al. 2021; Gibas et al. 2022). Similar approaches, including combinations of antiviral polymers and antiherpetic compounds, have shown improved efficacy against HSV (Yadavalli et al. 2020; Shankar and Alt 2014; Greeley et al. 2020). In line with this growing interest in combination therapies, our study aims to develop locally acting unit dose formulations of TDF and ZAD. Additionally, the study seeks to evaluate the potential combinatorial effects of these two drugs in vitro, with the goal of enhancing treatment efficacy against HSV-2 and mitigating the challenges associated with antiviral resistance and systemic side effects.

## Methods

### Vaginal film formulations

The films were prepared using the solvent casting technique. Composition of trial formulations is presented in Table 1. Polyvinyl alcohol (PVA) was weighed accurately and dispersed in the mixture of specified amounts of water (20%), glycerin (1%), and 2% polyethylene glycol 400 (PEG) under continuous stirring. ZA (0.49%) and TDF (0.41%) were separately dissolved in 30% of ethanol. The drug solutions were added to the polymer dispersion. The dispersion was magnetically stirred until a clear solution was obtained. The weight of the solution was made up with ethanol to 100%. The final solution was allowed to stand for an hour to remove the entrapped air bubbles and then was poured on a film-forming machine (VJ Instruments, Nagpur, India) with the help of the dispenser. The temperature of the film former was set at 80 °C. The film upon drying was carefully removed and cut into pieces of 5 cm length × 2 cm width size so that each piece would contain 12 mg of TDF and 10 mg of ZAD. The films were wrapped in aluminium foil and stored at 25 °C/65% RH for future studies.

**Table 1:** Composition of vaginal film formulation

Sr. no	Ingredients	Quantity (% w/w)					
		FE1	FE2	FE3	FE4	FE5	Placebo
1	TDF	0.49	0.49	0.49	0.49	-	-
2	ZAD	0.41	0.41	0.41	-	0.41	-
3	PVA	8	10	12	12	12	12
4	Glycerin	1	1	1	1	1	1
5	PEG 400	2	2	2	2	2	2
6	Distilled water	20	20	20	20	20	20
7	Ethanol	68.1	66.1	64.1	64.5	64.6	65

### Pessary formulations

The coco glyceride base was accurately weighed (35.87 g), transferred to a porcelain dish, and melted in a water bath at about 45 °C. Weighed quantities of TDF (72 mg) and ZAD (60 mg) were dispersed in a molten base with stirring (Table 2). The molten mass was poured into pre-lubricated suppository molds having six cavities and solidified at room temperature. The pessaries were removed from the molds and stored in aluminium foil sachets until further studies.

**Table 2:** Composition of pessary formulations

Ingredients	Quantity (% w/w)	
	F1	F2
TDF	0.49	0.49
ZAD	0.41	0.41
Hydrogenated coco glyceride grade 1	99.1	-
Hydrogenated coco glyceride grade 5	-	99.1

### Evaluation of films and pessaries

#### Appearance

The films and pessaries were visually inspected for overall appearance, color, homogeneity, and defects, if any.

#### Weight variation

Six films/pessaries were weighed individually and collectively. Average weight (*W*) and weight variation were calculated using following formula.

$$\text{Weight variation }({\%})=\frac{\text{Average weight}.-\text{Individual weight}.}{\text{Average weight}.}\times 100$$

#### Thickness

The thickness of the films was measured using a micrometre screw gauge (Mitutoyo, Japan). For each film, the thickness was measured at four corners and in the center. The mean of these values was considered one observation. The thickness of six films was measured in this manner (Apriliyani et al. 2020).

#### Folding endurance

The folding endurance of the films was determined by repeatedly folding a film at the same place until it was broken. The number of times the film folded at the same place without breaking was recorded as a folding endurance. The experiment was conducted in triplicate for each film formulation (Mishra et al. 2016).

#### Disintegration time

The films were added to 10 mL distilled water maintained at 37 °C. The water was stirred continuously at 50 rpm. The time required for films to dissolve completely was noted (Akil et al. 2011). Tablet disintegration test apparatus containing 700 mL of water maintained at 37 °C was used to determine the disintegration time of the pessary formulations. The time required for the suppositories to soften completely without any palpable core was recorded (Indian Pharmacopoeia). The experiment was performed on three samples of each formulation.

#### Moisture content (LOD)

The moisture content of the film formulations was determined using moisture analyser (ML-50, A&D Company Ltd., Japan). The film samples were placed in the analyzer set at 105 °C, and the loss on drying was recorded for three film samples (Apriliyani et al. 2020).

#### FT-IR studies

FT-IR spectra of TDF and ZAD; powdered formulations FE3, FE4, and FE5; and placebo film were recorded using a Bruker, Alpha II, Opus Software, USA, equipped with attenuated transmittance (ATR), covering a wavelength range of 500–4000 cm^−1^.

#### DSC studies

About 5 mg of pure TDF and ZAD; coarsely ground FE3, FE4, and FE5; and placebo film samples were subjected to DSC studies (DSC 1 Mettler Toledo, USA). An unfilled aluminum pan was used as a reference. DSC measurements were performed at a heating rate of 10 °C/min from 25 to 280 °C using an aluminum sealed pan. The sample cell was purged with nitrogen gas at 50 mL/min flow rate during the measurement. Pessary formulations F1 and F2 and placebo pessaries were also subjected to similar DSC studies.

#### Powder X-ray diffraction studies

TDF and ZAD; coarsely ground FE3, FE4, and FE5; and placebo film samples were subjected to powder X-ray diffraction studies (PXRD) studies. The samples were irradiated with monochromatic Cu-kα radiation and analyzed between 20° and 80° (2θ) employing a Rigaku Miniflex 600 X-ray diffractometer (Philips, Netherlands). The voltage and current used were 40 kV and 15 mA, respectively. The chart speed was 10 mm/s. PXRD studies were performed in the same manner for the pessary formulations.

#### Texture analysis

The films of formulation FE3 were tested for bursting strength and resilience in five replicates using a texture analyser (TA XT Plus equipped with Exponent software, Stable microsystems, UK). The film sample was securely placed on the sample platform. Aspherical probe (SMS HDP/FSR) equipped with a load sensor was employed to measure the bursting strength and resilience of the film. The movement of the probe was controlled by the micro stepper motor and monitored by the software. The probe after touching the film surface was moved at the speed of 1 mm/s into the film up to the distance of 5 mm. The distance travelled by the probe into the film until the film broke was considered resilience. The force required to break the film was regarded as bursting strength (Akil et al. 2011) which was measured by the load sensor attached to the probe. Similarly, pessary formulation F1 was subjected to evaluation of strength. Pessary formulation was placed on the sample platform. A 3-mm cylindrical probe (SMS P/3) was penetrated at a speed of 0.5 mm/s and up to a 3-mm distance into the pessary. The force necessary to break the suppository was considered the pessary’s hardness. The force was measured by the load sensor attached to the probe.

#### Drug content

The film formulation FE3 was cut into small pieces, ground coarsely in a mortar, and dispersed in 25 mL of purified water. The dispersion was sonicated for 10 min and filtered using nylon filter (0.45 µm). Pessary formulations were also processed similarly for the determination of the drug content. The filtrates were appropriately diluted and subjected to complexometric titration for the estimation of ZAD and RP-HPLC for the analysis of TDF. The analytical methods are mentioned as follows.

USP procedure with slight modification was followed for quantifying ZAD in the formulation. For the filtrate, 1 mL was transferred to a 250-mL volumetric flask. For the nitric acid (3.0 M), 10 mL was added to the flask and warmed on a hot plate for a few minutes. The solution was diluted with deionized water to exactly 250 mL. The sample of 25 mL was pipetted out from the above solution, and 25 mL of deionized water was added to a graduated cylinder. The pH of the solution was adjusted to 10 using a concentrated ammonia solution. A few drops of Eriochrome Black T indicator solution were added. The solution was titrated against 0.005 M EDTA solution until the colour of the solution changed from purple to pure blue. ZAD content was determined using following factors. Each milliliter of 0.005 M EDTA is equivalent to 1.098 mg of ZAD (United States Pharmacopoeia).

RP HPLC system (e2695, Waters, USA) with UV detector (2489, Waters, USA) was employed to determine TDF content. The mobile phase comprising acetonitrile:water (60:40) was filtered through 0.45-µ nylon filter and run through C_18_ column (150 mm × 4.6 mm ID, 5 µm, Zorbax, USA) at the flow rate of 0.5 mL/min. The detector was set at the wavelength of 260 nm and sample size of 20 µL was analyzed at ambient column temperature (Hoang et al. 2019; Kandagal et al. 2008).

#### In vitro drug release studies

Film formulation FE3 was added to the beaker containing 25 mL simulated vaginal fluid (SVF) (Shapiro et al. 2022). The contents of the beaker were subjected to gentle agitation at 37 ± 0.5 °C by placing the beaker in an orbital shaker cum incubator (Biomedica, Mumbai, India). At 15-, 30-, 45-, 60-, and 90-min time points, 2.5-mL aliquots were withdrawn and replaced with fresh 2.5 mL of SVF. The ZAD and TDF concentrations in samples were analyzed using complexometric titration and RP- HPLC method mentioned in the previous section. The release studies were performed similarly for the pessary formulations.

### Virus, cells, and test compounds

The virus strain, Human Herpes Simplex Virus 2 (Strain G,VR-734, ATCC, Manassas, VA, USA), used for the study was obtained from ATCC. African green monkey kidney cells (Vero) (ATCC ®CCL 81^TM^ strain) were used for virus propagation and plaque assay. Minimal Essential Medium (MEM) (Gibco; Thermo Fisher Scientific, Inc.) was used for cell culturing. Media was supplemented with 10% fetal bovine serum (FBS) (Gibco; Thermo Fisher Scientific, Inc.). ZAD (99.12% purity) was purchased from Avra Synthesis Pvt. Ltd. (Hyderabad, India), and TDF (98% purity) was purchased from Laurus Labs (Andhra Pradesh, India). The stock solutions of the drug formulations (10 mM) were prepared by diluting 2.19 mg of ZAD and 6.35 mg of TDF in 1 mL of distilled water.

### Cytotoxicity assay

The cytotoxicity of the drug formulations was assessed using crystal violet staining. Vero cells were seeded in a 96-well plate (3 × 10^5^ cells/mL) and incubated at 37 °C and 5% CO_2_ for 48 h. Different drug concentrations of ZAD and TDF were prepared in MEM-maintenance media (MM) ranging from 1.9 to 1000 µM for ZAD and 62.5 to 1500 µM for TDF. Additionally, combinations of ZAD and TDF were prepared in equimolar ratio, with concentrations ranging from 2.5 to 500 µM. Each concentration was added in triplicate to the confluent cell monolayer and subsequently incubated for 48 h. Periodic microscopic observations were made at different time points to assess cytotoxicity-induced morphological changes. After 48 h, cells were fixed with 70% v/v methanol followed by 0.5% w/v crystal violet staining. The stained plates were subjected to spectrophotometric analysis using a microplate reader (ELx800 BioTek Instruments, Inc., Highland Park, Winooski, USA) at 490 nm, and the absorbance values were recorded. Percentage cytotoxicity was calculated using the formula:

$$\text{Percentage cytotoxicity}= \frac{\text{Absorbance control}-\text{Absorbance test}}{\text{Absorbance control}}\times 100$$

### HSV-2 quantification by plaque assay

A 12-well plate seeded with 1.25 × 10^5^ cells/mL (2 mL per well) was used for the experiment. The media was decanted, and the wells were rinsed using 100 μL MEM MM. Two hundred microliters of MEM MM were added to the rinsed wells, and 100 μL of tenfold diluted (10^−1^ to 10^−6^) virus was added to the respective wells in triplicates. The plate was then incubated for 1 h at 37 °C and 5% CO_2_ for virus adsorption. Post incubation, an agarose overlay (2% w/v agarose 2 × -yeast extract lactalbumin hydrolysate and 7% v/v sodium bicarbonate; 1.5 mL) was added to each well and incubated for 24 h at 37 °C and 5% CO_2_. After incubation, a second agarose overlay (1.5 mL) with an additional 0.3% v/v neutral red stain was added to the wells to visualize plaques. Twenty-four hours after the addition of the second overlay, the number of plaques in each dilution was counted under a white light source, and virus was quantified in terms of plaque forming unit (PFU)/mL.

### Plaque reduction assay

A 12-well plate seeded with Vero cells at a 1.25 × 10^5^ cells/mL density was used for plaque reduction assay. A standard viral dose of 100 PFU was used to infect the cell monolayer. ZAD at concentrations of 30 µM and 100 µM, and TDF at concentrations of 10 µM, 100 µM, and 400 µM, were evaluated individually. Additionally, for the ZAD-TDF combination study, equimolar concentrations of 50 µM and 100 µM were assessed. Both individuals and combinations underwent three treatments: post-infection, co-infection, and pre-infection. Acyclovir (ACV), the standard antiviral drug, was used as a positive control (8.9 µM) in all the assays (Weinberg et al. 1992).

#### Post-infection treatment

Virus inoculum was added to the cells and incubated for 1 h. Post removal of the inoculum, drugs were added.

#### Co-infection treatment

Virus and drug dilutions were incubated for 1 h and immediately used to infect the cells for 1 h. The mixture was removed, and fresh media was added.

#### Pre-infection treatment

Drug concentrations were added to the cells and incubated for 1 h. Virus inoculum was then added and incubated for another hour, followed by removal of the mixture and fresh media addition.

Following all three treatment protocols, an agarose overlay of 1.5 mL was added to each well and incubated for 24 h at 37 °C and 5% CO_2_. This was followed by a second overlay (1.5 mL) addition and counting of plaques, as mentioned in the plaque assay protocol above. The percentage of reduction in plaques was calculated using the formula:

$$\mathrm{Percentage}\, \mathrm{of}\, \mathrm{reduction}\, \mathrm{in}\, \mathrm{plaques}= \frac{\mathrm{Number}\, \mathrm{of}\, \mathrm{plaques}\, \mathrm{in}\, \mathrm{virus}\, \mathrm{control}- \mathrm{Number}\, \mathrm{of}\, \mathrm{plaques}\, \mathrm{in}\, \mathrm{test}}{\mathrm{Number}\, \mathrm{of}\, \mathrm{plaques}\, \mathrm{in}\, \mathrm{virus}\, \mathrm{control}} \times 100$$

Virus control—wells with 100 PFU of virus and no drugs. Test—wells with 100 PFU of virus with different concentrations of drugs.

### Statistical analysis

The data from the plaque reduction assays are expressed as the mean ± SEM. A one-way analysis of variance (ANOVA) was followed by Dunnett’s multiple comparisons tests, which was performed using GraphPad Prism (version 10.2.3). A “*p*” value of less than 0.05 was considered statistically significant. The CC_50_ and EC_50_ values were computed using log(inhibitor) vs. normalized response-variable slope dose–response models using GraphPad Prism (version 10.2.3). The combination index (CI) was calculated using Chou and Talalay’s multiple drug effect equation using the CompuSyn algorithm (https://www.combosyn.com). CI values < 1, = 1, and > 1 indicate synergism, additive effect, and antagonism, respectively (Chou and Talalay 1984).

## Results

### Vaginal film formulations

Various polymers like hydroxypropyl methylcellulose 100 Cps (Methocel K 100 LV, Dow Chemicals), polyvinyl pyrrolidone K 30, ethyl cellulose, and PVA 4–88 were explored for the development of film formulation. The trial runs of the placebo films using these excipients have been shown in Supplementary Table 1. The resulting films were evaluated for appearance, weight variation, thickness, folding endurance, and disintegration time. Polyvinyl alcohol films demonstrated excellent clarity, appearance, homogeneity, soft and flexible texture, and physical strength (Table 3). These observations were in agreement with reported literature (Akil et al. 2015; Gong et al. 2017). Table 1 shows formulation trials incorporating varying proportions of PVA while keeping the concentration of plasticizers viz. glycerin and PEG 400 constant. At 8 and 10% concentrations of PVA in formulation FE1 and FE2, the resulting films were thinner and exhibited the folding endurance of less than 500. Ease of handling of the films during manufacturing, packing, and administering is crucial for the commercial and clinical success of a film formulation (Takeuchi et al. 2020). A robust and flexible film formulation with higher folding endurance was thus the goal of the film formulation development. Formulation FE3 with 12% PVA resulted in the film of the desired thickness, folding endurance of > 500, and disintegration time. Hence, formulation FE3 was chosen for further studies (Figure S1a). Formulations FE4 and FE5, and placebo, had a similar composition to FE3 except that they were made without either of the drugs or both. The moisture content of the formulations was less than 10% w/w, which was within the acceptance criteria (Patel et al. 2023).

**Table 3:** The physical characteristics of the film formulations

No	Parameters	FE1	FE2	FE3	FE4	FE5	Placebo
1	Appearance	+	** + **	** + **	** + **	** + **	** + **
2	Average weight^#^ (mg)	232.5 ± 9.4	245.1 ± 11.2	258.7 ± 11.7	253 ± 14.4	261 ± 12.4	251 ± 13.4
3	Thickness (µm)^#^	195 ± 14	220 ± 16	234 ± 12	231 ± 11	235 ± 15	229 ± 15
4	Folding endurance^#^	348 ± 10	421 ± 8	500 +	500 +	500 +	500 +
5	Disintegration time	3 min 20 s	4 min 40 s	5 min 25 s	5 min 17 s	5 min 10 s	5 min 26 s
6	Moisture content^#^ (%)	6.25 ± 0.6	6.48 ± 1.1	7.06 ± 0.5	6.56 ± 0.4	6.72 ± 0.4	6.05 ± 0.2

“ + ” transparent, homogeneous, flexible, soft, without any defects

^#^Data are mean ± SD of *n* = 6

#### FT-IR studies

**Fig. 1:**
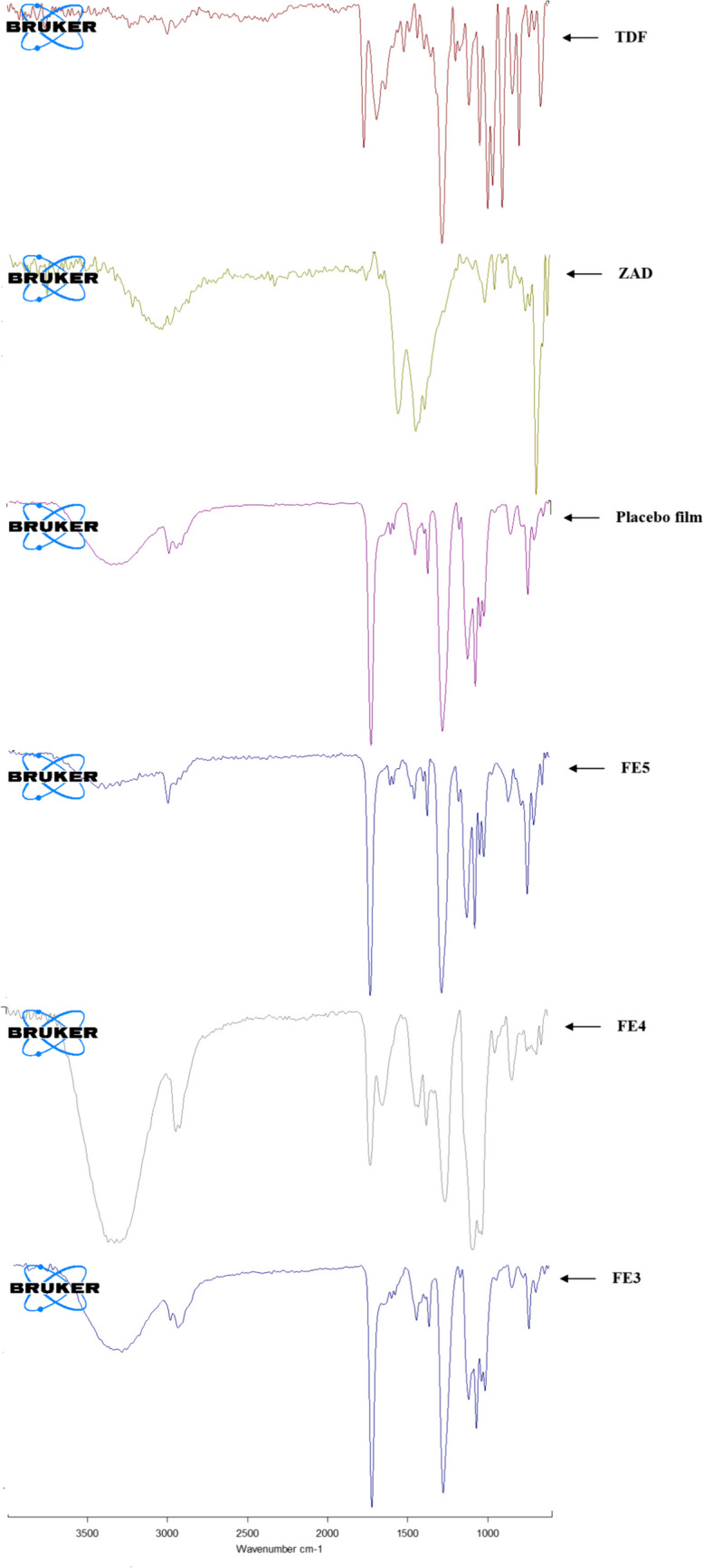
FTIR spectra of TDF, ZAD, placebo film, FE4 (TDF film), FE5 (ZAD film), and FE3 (ZAD + TDF) film

Formulations FE3, FE4, and FE5 and placebo film were subjected to FTIR studies to assess any incompatibility between individual drugs and film-forming excipients. Similarly, the IR spectrum of FE3 would indicate interaction, if any, between two drugs (Fig. 1). The characteristic peaks of ZAD, such as carbonyl stretching (1763 cm^−1^), C–O stretching (1443 cm^−1^), C–CH3 stretching (1056 cm^−1^), and CH3 asymmetric bending (1078 cm^−1^), were present in formulation FE5 and FE3. Similarly, P = O stretching at 1170 cm^−1^, N–H bending seen at 1622 cm^−1^, carbonyl stretching (1763 cm^−1^), and imine stretching vibration frequencies (1674 cm^−1^) of TDF were retained in the spectra of the formulations FE3 and FE4. The broad peaks in the 3200 to 3500 cm^−1^ range observed in all the film formulations represented overlapping of OH group stretching frequencies contributed by both the drugs along with PVA and NH_2_ vibrations of TDF. The IR spectra of formulations thus confirmed the absence of any interaction between the drugs and among the drugs and the film formulation excipients.

#### DSC studies

Thermographs of neat TDF and ZAD, and formulations FE3, FE4, and FE5, and placebo film are depicted in Figure S2. The thermogram of TDF shows a sharp melting endotherm in the range of 118 to 122 °C, indicating the crystalline nature of the drug. Similarly, endotherm in the range of 249 to 252 °C in the thermogram of ZAD correlates well to its melting point. A broad endotherm in the temperature range of 80 to 120 °C indicates loss of water of crystallinity of ZAD. Placebo film exhibited a small and broad endotherm in the 180 to 200 °C range, which means melting PVA with decomposition (Tsioptsias et al. 2023). A broad endotherm in the 80 to 120 °C range could be a combination of the glass transition temperature of PVA and loss of moisture from the film sample. The film formulations EF3 (containing both TDF and ZAD), EF4 (containing only TDF), and EF5 (containing only ZAD) show similar thermal events as in the case of placebo film. The absence of melting endotherms of either drug indicated their crystallinity loss in the film formulations.

#### PXRD study

The X-ray diffractograms of pure TDF and ZAD exhibited intense peaks of crystallinity. In contrast, the film formulations containing either a single drug or both the drugs in combination showed a complete absence of peaks projecting amorphization of the drugs in the formulations (Figure S3). The PXRD results corroborated sufficiently with DSC study observations.

#### Texture analysis

Formulation FE3 was subjected to texture analysis to evaluate bursting strength and resilience. The bursting strength is proportional to the compression force that needs to be applied to break the film. The force of 1620 ± 125 g required to break the film of formulation FE3 indicated its adequate mechanical strength that can sustain the stress during handling, processing, packaging, and shipping (Figure S4). Film resilience is the maximum distance a film is stretched before the first fracture appears. It indicates the flexibility of the film. The resilience of 3.63 ± 0.11 mm measured by the texture analyzer confirmed the satisfactory flexibility of FE3 formulation. The higher folding endurance (> 500) of the film also confirmed reasonable flexibility of the film. The data of bursting strength and the resilience of the film formulation was in agreement with the earlier reported studies (Akil et al. 2011; Gong et al. 2017; Kumar et al. 2013).

#### Drug content

The ZAD and TDF content of the formulation of FE3 was 99.65 ± 1.2% and 96.43 ± 1.8%, respectively, which was well within the acceptance limits of 95 to 105% (Patel et al. 2023).

#### In vitro drug release

**Fig. 2:**
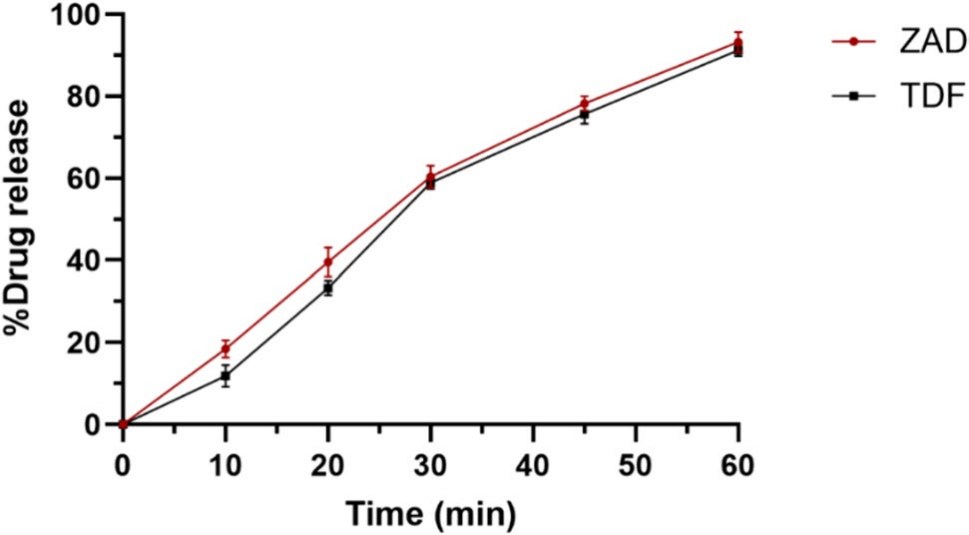
In vitro drug release studies of film formulation FE3 (Data shown are mean ± SD of *n* = 6)

Formulation FE3 exhibited more than 90% release of ZAD and TDF over 60 min (Fig. 2). Using PVA, a water-soluble polymer, as a film former resulted in faster disintegration of the formulation in aqueous medium. Reasonable aqueous solubility of both the drugs and loss of crystallinity in the film formulation could be the contributing factors for their augmented dissolution rate.

### Pessary formulation

Placebo pessary formulations were prepared using glycerol-gelatin base, polyethylene glycol 4000 and 6000 bases, and coco glyceride bases. The pessaries were observed for appearance, consistency, smoothness, defects, etc. (data not shown). Coco glyceride–based pessaries scored well in all these aspects compared to other bases, and hence, coco glyceride grade 1 and 5 bases were chosen for the formulation development (Figure S1b). Evaluation of formulations F1 is shown in Table 4.

**Table 4:** Evaluation of pessary formulations

No	Appearance^#^	Weight variation^#^ (g)	Disintegration time*	Drug content* (%)
F1	White, smooth, with no visual defects	1.14 ± 0.24	11 min 20 s	ZAD 93.5 ± .2.1 TDF 95.2 ± .1.8
F2	White, smooth, with no visual defects	1.17 ± 0.46	20 min 19 s	ZAD 94.8 ± .2.3 TDF 94.3 ± .2.5

^#^The values are mean of *n* = 6 ± SD

^*^The values are mean of *n* = 3 ± SD

#### DSC and PXRD studies

Thermograms of placebo formulations comprising coco glyceride grade 1 and 5 exhibited endothermic peaks at 35 to 45 °C, indicating their melting (Figure S5). The absence of melting endotherms of TDF and ZAD in the thermograms of formulations F1 and F2 indicated their amorphization in the formulations. These results were confirmed by PXRD studies (Figure S6), wherein intense peaks of crystallinity of both drugs were absent in the diffractograms of formulations F1 and F2.

#### Texture analysis

Pessaries of formulation F1 were further subjected to texture analysis. The strength, also known as the hardness of pessaries, was observed to be 340.5 ± 40.2 g (Figure S7). Break strength is the maximum force required to break the product into pieces. Since the product develops small fissures before completely giving way, smaller dips in the graphs can be seen. These could be considered as a measure of the structural uniformity of the product. The hardness of 340 g indicates satisfactory mechanical strength of the formulation to withstand the stresses during packaging, shipping, and handling.

#### In vitro drug release studies

**Fig. 3:**
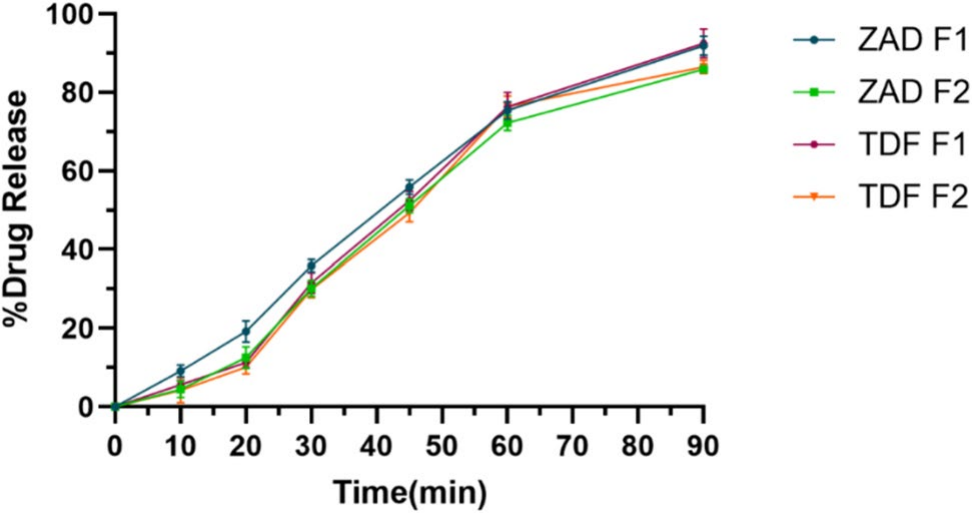
In vitro drug release from pessary formulations (values shown are mean ± SD of *n* = 6)

Formulations F1 and F2, when subjected to in vitro drug release studies, exhibited about 92% and 86% drug release respectively at the end of 90 min (Fig. 3). The lower dissolution rate of formulation F2 compared to F1 at initial time points could be attributed to its longer disintegration time. Unlike films, slower dissolution rates of pessary formulations compared to film formulation could also be attributed to slower disintegration of the pessary.

### Evaluation of cytotoxicity of ZAD, TDF, and ZAD-TDF combination

**Fig. 4:**
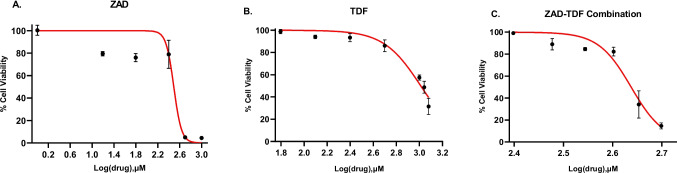
Effect of **A** zinc acetate dihydrate (ZAD), **B** tenofovir disoproxil fumarate (TDF), and **C** ZAD-TDF combination on Vero cells (3 × 10^5^ cells/mL). Each data point represents the mean of OD (optical density) values (*n* = 3) ± SEM

The cytotoxicity profiles of ZAD and TDF were evaluated individually in Vero cells using Crystal Violet staining. No cytotoxicity was observed until 250 µM for ZAD and until 500 µM for TDF. The CC_50_ values for ZAD and TDF were determined to be 310 µM and 1027 µM, respectively. No cytotoxicity was observed until 250 µM for the ZAD-TDF combination. CC_50_ for the combination was 434 µM (Fig. 4).

### Evaluation of individual antiviral activity of ZAD and TDF

Non-cytotoxic concentrations, 30 and 100 µM viral of ZAD, and 10, 100, and 400 µM of TDF were assessed for antiviral activity.

**Fig. 5:**
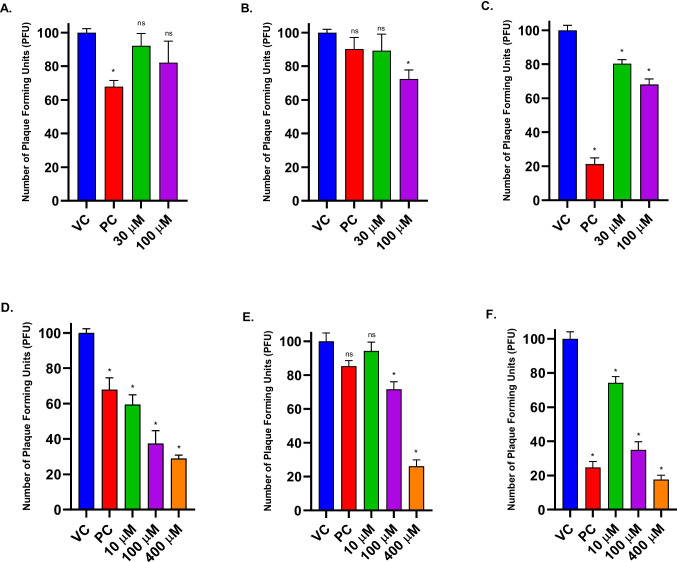
HSV-2 plaque reduction profile of zinc acetate dihydrate (**A**, **B**, **C**) and tenofovir disoproxil fumarate (**D**, **E**, **F**) in pre-infection (**A**, **D**), co-infection (**B**, **E**), and post-infection treatments (**C**, **F**). Each bar represents the average number for plaques (*n* = 3) at different drug concentrations. The results are expressed as mean ± SEM. Asterisks indicate a significant difference (*p < 0.05) in comparison with the virus control group (VC = 100 PFU/mL). Acyclovir was used as a positive control (PC)

Both the concentrations of ZAD did not show any significant antiviral activity in pre-infection treatment (Fig. 5A). In post-infection treatment, ZAD exhibited moderate inhibition, approximately 20% at 30 µM and 30% at 100 µM (Fig. 5C). In co-infection treatment, at 30 µM, ZAD did not exhibit any significant reduction, but at 100 µM about 30% inhibition was observed (Fig. 5B).

All three concentrations tested for TDF in all the treatments showed significant antiviral activity, except 10 µM in co-infection treatment (Fig. 5D, E, F). EC_50_ of TDF in pre-infection treatment was determined to be 21.6 µM. In co- and post-infection treatments, the EC_50_s were 191.2 µM and 43.3 µM, respectively. In pre- and post-infection, 30–40% reduction in infectivity was observed at the lowest concentration tested (10 µM), whereas more than 70% inhibition was observed at the highest concentration tested (400 µM), across all three treatments.

### Evaluation of combinatorial antiviral activity of ZAD and TDF

**Fig. 6:**
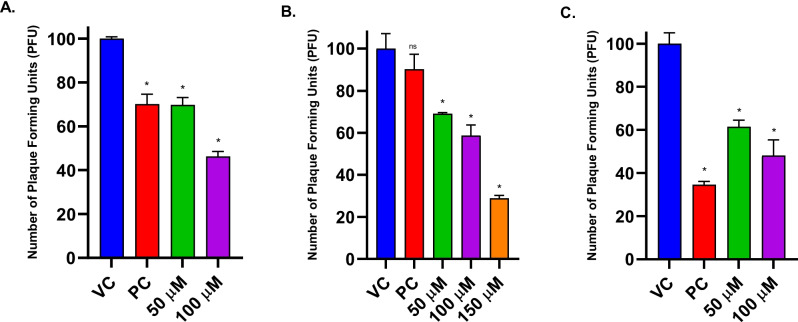
HSV-2 plaque reduction profile of ZAD-TDF combination. **A** Pre-infection, **B** co-infection, **C** post-infection treatments. Each bar represents the average number of plaques (*n* = 3) at different drug concentrations. The results are expressed as mean ± SEM. Asterisks indicate a significant difference (*p < 0.05) in comparison with the virus control group (VC = 100 PFU/mL). Acyclovir was used as a positive control (PC)

The antiviral efficacy of the combination was evaluated using non-cytotoxic concentrations of 50 to 150 µM of ZAD and TDF. The combination demonstrated significant inhibitory effects across the three treatments at all concentrations. In the pre-, co-, and post-infection treatments, EC_50_s were 89 µM, 99 µM, and 90 µM, respectively (Fig. 6A, B, C). CompuSyn software was utilized to compute CI values for the combination. In both post- and pre-infection treatments, the combination showed CI of 1.1 and 2.1 at the 50% inhibitory concentration. The combination showed a CI of 0.4 at the 50% inhibitory concentration in co-infection treatment.

### Selectivity indices

Selectivity index (SI) (Table 5) was calculated for the TDF and ZAD-TDF combination as the ratio between cytotoxicity and antiviral activity by dividing the CC_50_ value by the EC_50_ value (CC_50_/EC_50_).

**Table 5:** Selectivity indices of TDF and ZAD-TDF combination

DRUG	CC_50_	TREATMENT	EC_50_	SI
TDF	1027 µM	Pre-infection	21.6 µM	47.5
		Co-infection	191.2 µM	5.3
		Post-infection	43.3 µM	23.7
ZAD-TDF combination	434 µM	Pre-infection	89.9 µM	4.8
		Co-infection	99.1 µM	4.3
		Post-infection	90.5 µM	4.7

## Discussion

Though vaginal formulations entailing TDF and ZAD in combination have not been reported so far in the literature, vaginal rings and film formulations of TDF in combination with emtricitabine have been proposed earlier (Srinivasan et al. 2016; Moss et al. 2014; Cautela et al. 2019). Recently, pessary formulations of TDF-loaded sodium alginate microspheres have been reported by Avlani and co-workers (Avlani et al. 2024). The same group has earlier developed a dispersible tablet formulation of TDF for vaginal application (Avlani et al. 2023). Unit dose formulations such as films, pessaries, and tablets ensure uniform and reproducible local administration of drugs. These are advantageous over creams, gels, or ointments, wherein inconsistent and variable drug exposure is often encountered (Mesquita et al. 2012). Our work involved the development of films and pessaries, which also are expected to avoid leakage of the formulation upon vaginal administration, unlike creams and gels. Further, the bullet or torpedo shape of the pessary allows for ease of insertion in the vaginal cavity (Ham et al. 2017), whereas a film can be rolled and administered conveniently (Notario-Pérez et al. 2020). Our work aimed to produce immediate-release film and pessary formulations, which upon vaginal application would provide higher drug concentration in the vulvovaginal region that is expected to offer more excellent antiviral protection during or immediately post-coitus. Pessary formulation would also allow rectal administration for providing prevention of HSV-2 infection during anal sex.

The development of film formulation involved initial trials to select the film-forming polymer and plasticizer. Evaluation of placebo films’ appearance, texture, thickness, folding endurance, and disintegration time led us to choose PVA as a film former. Additional merits of PVA, such as its safe, biocompatible, and mucoadhesive nature, were considered during its selection for formulation development (Akil et al. 2015; Machado et al. 2016; Cunha et al. 2014). The optimized formulation containing 12% PVA, 2% PEG 400, and 1% glycerin released more than 90% of TDF and ZAD within 1 h, which was desirable. Pessary formulations were developed using two grades of hydrogenated coco glycerides. These grades are glycerides of hydrogenated coconut oil with different hydroxyl values and differ slightly in their melting points. Grade 1 has a melting point of 34–35 °C, whereas grade 5 melts from 36 to 37 °C. Both the grades comply with the compendial monograph of hard fats. Longer disintegration times of formulations could be attributed to slow melting and softening of the water-insoluble fatty bases at 37 °C. The higher melting temperature of the grade 5 base delayed the disintegration time of formulation F2 to over 20 min compared to 11 min in the case of formulation F1, comprising grade 1 base. Longer disintegration times of pessaries retarded their drug release to some extent in comparison with that of film formulations. Pessaries of formulation F1 exhibited more than 90% release of ZAD and TDF at 90 min, unlike film formulation FE3, which took 60 min to release more than 90% of incorporated drugs. Almost similar release rates of both the drugs from either of the dosage forms are expected to work in tandem and provide synergistic action. These unit dose formulations of TDF and ZAD are expected to provide prophylaxis of genital herpes infection when administered before or just after coitus.

To investigate potential combinatorial effects of TDF and ZAD, we performed cytotoxicity assays and antiviral activity testing. Cytotoxicity profiling of compounds demonstrated the pronounced cytotoxic effects of ZAD in vitro. Zinc and its salts pose challenges in vitro testing due to their inherent toxicity to cells in two-dimensional (2-D) cell culture systems (Fernández-Romero et al. 2012). However, findings from in vivo investigations have indicated zinc acetate to be both non-toxic and efficacious against HSV-2 infection (Bourne et al. 2005). In this study, the non-cytotoxic concentrations of ZAD tested for antiviral activity were substantially lower than those employed in active concentrations, as observed in clinical and animal experimentation. The evaluated concentrations of ZAD exhibited moderate inhibitory effects against HSV-2 infection, achieving a maximum of ~ 30% inhibition in post and co-infection treatments. Zinc and its salts have been proven to inhibit penetration and egress stages of the virus life cycle (Kümel et al. 1990; Marreiro et al. 2021). This could potentially be the reason for the moderate inhibition by ZAD in co- and post-infection. When compared to ZAD, TDF was less cytotoxic and had better antiviral activity against HSV-2, as indicated by its EC_50_ values across the treatments. TDF has been proven to inhibit HSV-DNA polymerase in both in vitro and in vivo setups (Nixon et al. 2014). Inhibitors of DNA polymerase, such as nucleoside/nucleotide analogues, generally show potent antiviral activity in pre and early-post-infection treatments (Goulding et al. 2022). This was also evident in the case of the positive control (ACV) used, which showed the lowest levels of inhibition in the co-infection treatment (9–14%) at 8.9 µM compared to a much higher inhibition in pre-infection (30–35%) and post-infection (65–80%). Since approved drugs, like acyclovir, are usually the choice of control in antiviral assays related to HSV, we selected it for our study to derive a reliable baseline for comparison. The varying antiviral activity of acyclovir across the three treatments was consistent among the drug regimens (ZAD, TDF, and ZAD-TDF), validating the methodology of our experimental design.

Since ZAD and TDF exhibited different modes of action against HSV-2 infections, we sought to explore if their combinatory effects would potentially be synergistic. Even though the combination could reduce infectivity in post and pre-infection by 50% at ~ 90 µM, much lower concentrations of TDF alone (21.6 µM and 43.3 µM) were enough for the same reduction. On the contrary, in co-infection treatment, the combination reduced the concentration required for 50% inhibition by 90 µM compared to TDF alone (191.2 µM). These observations were verified using CompuSyn software, where CI values indicated evident antagonism between ZAD and TDF in post and pre-infection treatment but synergism in the case of co-infection treatment. Targeting different stages of the viral life cycle, including penetration and egress (ZAD) and replication (TDF), could enhance collective efficacy in co-infection treatment. The selectivity indices of the combination were lower than those of the selectivity indices of TDF across all treatments. These reductions in the values of selectivity indices can be solely attributed to the reduction of CC_50_ of combination due to the high cytotoxicity of ZAD in cell culture systems. This is not the case in vivo wherein administration of ZAD-carrageenan gel with the highest concentration of 1.5% ZAD did not lead to any injury to the mucosal lining of cervicovaginal and rectal areas of mice (Fernández-Romero et al. 2012). Similarly, phase 1 clinical trials of in healthy women involving vaginal application of 4 mL of 1% ZAD gel for the period of 14 days confirmed excellent safety and tolerability of the formulation (Friedland et al. 1999). Further, the in silico computation of CI is only indicative of possible interactions between the antiviral activities of the two drugs and hence needs validation by performing the studies in a suitable animal model of HSV.

## Conclusion

Our study suggests a potential synergy between zinc acetate dihydrate (ZAD) and tenofovir disoproxil fumarate (TDF) against HSV-2 infections in vitro. Despite cytotoxicity challenges, ZAD at non-toxic levels displayed moderate inhibition, while TDF exhibited significant antiviral activity with lower toxicity. Combining these drugs, each targeting HSV-2 through distinct mechanisms, showed promise, especially in co-infection treatment, where it significantly reduced the TDF concentration needed for inhibition. Film and pessary formulations containing TDF and ZAD were developed with satisfactory physicochemical properties, texture, and desired in vitro drug release profiles. These unit dose formulations are expected to deliver consistent and predictable doses of TDF and ZAD in vulvovaginal and/or rectal regions to provide prophylaxis of HSV-2 infection. However, preclinical pharmacokinetics, safety, and efficacy studies are essential before the formulations can be tested for clinical translation.

## Supplementary Information

Below is the link to the electronic supplementary material.

Supplementary file1 (DOCX 3371 KB)

## Data Availability

Data is provided within the manuscript or supplementary information files.

## Abbreviations

HSV-2: Herpes simplex virus-2
CC_50_: 50% Cytotoxic concentration
EC_50_: 50% Effective concentration
UL: Unique long
PRA: Plaque reduction assay
ZAD: Zinc acetate dihydrate
TDF: Tenofovir disoproxil fumarate
FDA: Food and Drug Administration
VZV: Varicella zoster virus
HIV: Human immunodeficiency virus
HBV: Hepatitis B virus
HCV: Hepatitis C virus
ATCC: American Type Culture Collection
CPE: Cytopathic effect
PFU: Plaque forming units
SI: Selectivity index
CI: Combination index
FTIR: Fourier transform infrared spectroscopy
DSC: Differential scanning calorimetry
PXRD: Powder X-ray diffraction
