# Correction: Development and characterization of formulations based on combinatorial potential of antivirals against genital herpes

**DOI:** 10.1007/s00210-024-03668-6

**Published:** 2024-12-05

**Authors:** Mahesh Gaikwad, Amal George, Aparna Sivadas, Kavitha Karunakaran, Sudheesh N, Siddappa N. Byrareddy, Chiranjay Mukhopadhyay, Piya Paul Mudgal, Madhur Kulkarni

**Affiliations:** 1 SCES’s Indira College of Pharmacy, New Mumbai Pune Highway, Tathawade, Pune, India; 2 Manipal Institute of Virology, Manipal Academy of Higher Education, Manipal, India; 3 Department of Pharmacology and Experimental Neuroscience, University of Nebraska Medical Centre, Omaha, NE USA

**Correction: Naunyn-Schmiedeberg's Archives of Pharmacology**

10.1007/s00210-024-03468-y

In the published original paper, the name of the 6th author, Dr. Siddappa N. Byradeddy, has been misspelled.

The correct name is Siddappa N. Byrareddy.

