# Supplementary material for: Development and characterization of formulations based on combinatorial potential of antivirals against genital herpes
Source: Naunyn Schmiedebergs Arch Pharmacol. 2024 Sep 30;398(3):3103–17. doi: 10.1007/s00210-024-03468-y (PMC11919951; doi:10.1007/s00210-024-03468-y)
Supplement: Supplementary file 1 — Supplementary file1 (DOCX 3371 KB) [file 210_2024_3468_MOESM1_ESM.docx]

**Supplementary Table 1:** Placebo film formulations using various polymers

| **Ingredients**  **(%w/w)** | **FA** | **FB** | **FC** | **FD** | **FE** | **FF** | **FG** | **FH** | **FI** | **FJ** | **FK** |
| --- | --- | --- | --- | --- | --- | --- | --- | --- | --- | --- | --- |
| HPMC K 100 LV  (polymer) | 3 | 3 | - | - | - | - | 2 | - | - | 2 | - |
| PVP K-30  (Polymer) | - | 1 | - | - | - | 2 | - | 2 | 2 | - | - |
| PVA 4-88  (Polymer) | - | - | 4 | 6 | 8 | 6 | 6 | 6 | 3 | 6 | 8 |
| PEG 400  (Plasticizer) | 2 | 2 | 2 | 2 | 2 | 2 | 2 | 2 | 2 | 2 | 2 |
| Glycerin  (Plasticizer) | 1 | 1 | 1 | 1 | 1 | 1 | 1 | - | - | 1 | 1 |
| Dibutyl phthalate  (Plasticizer) | - | - | - | - | - | - | - | 2 | 4 | - | - |
| Distilled  water  (Solvent) | 20 | 20 | 20 | 20 | 20 | 20 | 20 | 20 | 20 | 20 | 20 |
| Ethanol  (Solvent) | 74 | 73 | 73 | 71 | 69 | 69 | 69 | 68 | 69 | 69 | 69 |

**
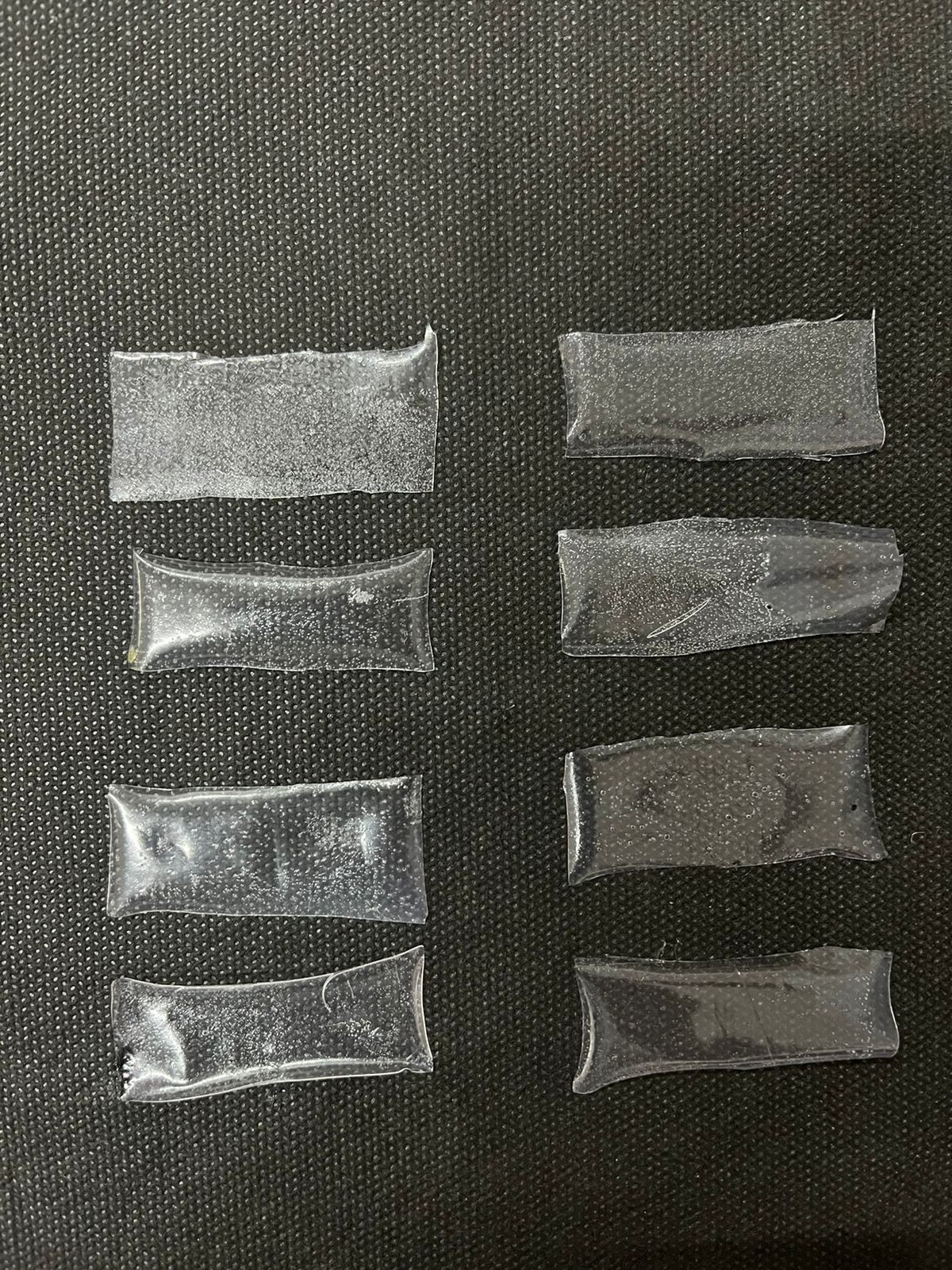
**

**Figure S1a: FE3 Film formulation containing TDF and ZAD**


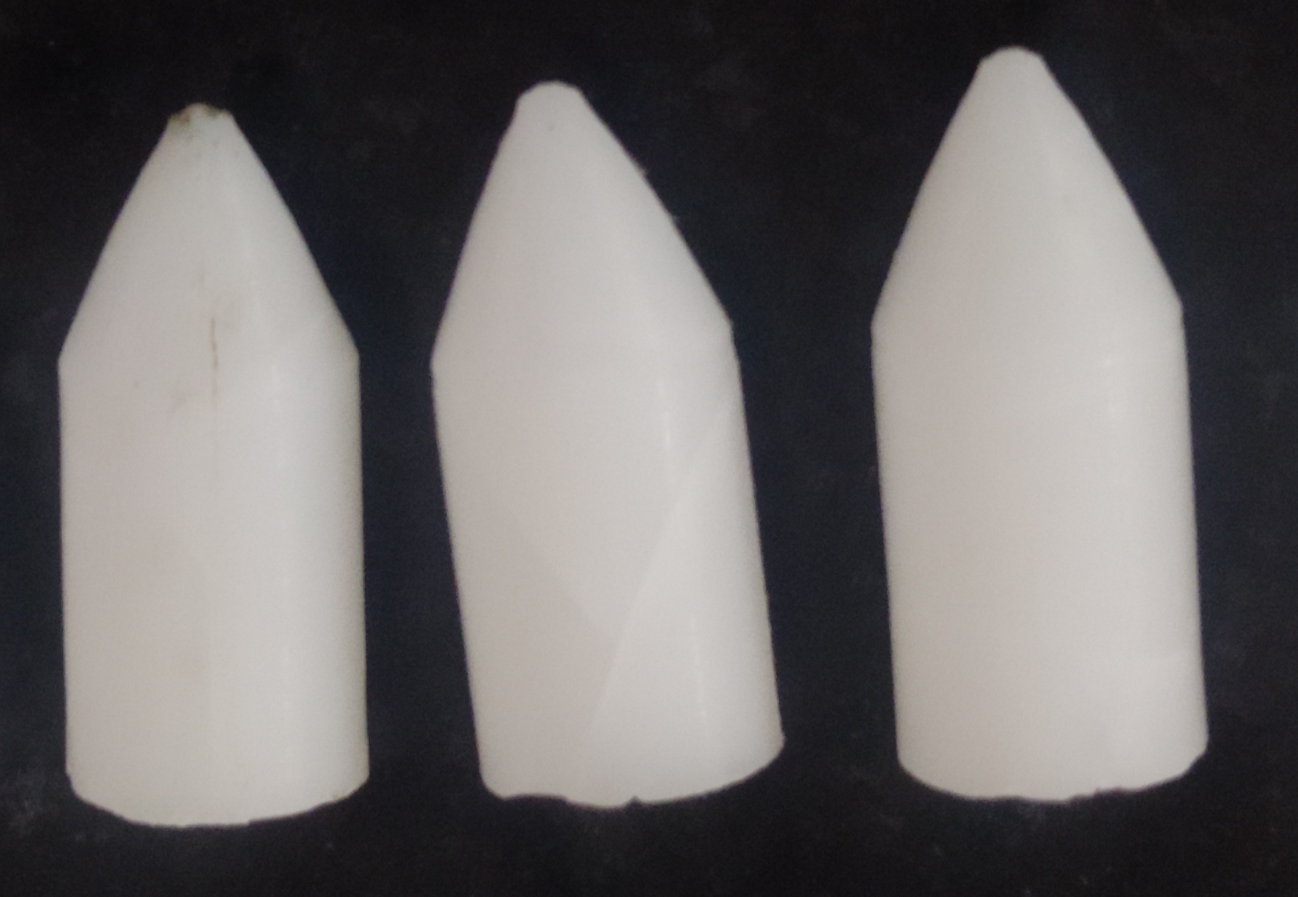


**Figure S1b: F1 Pessary formulation containing TDF and ZAD**


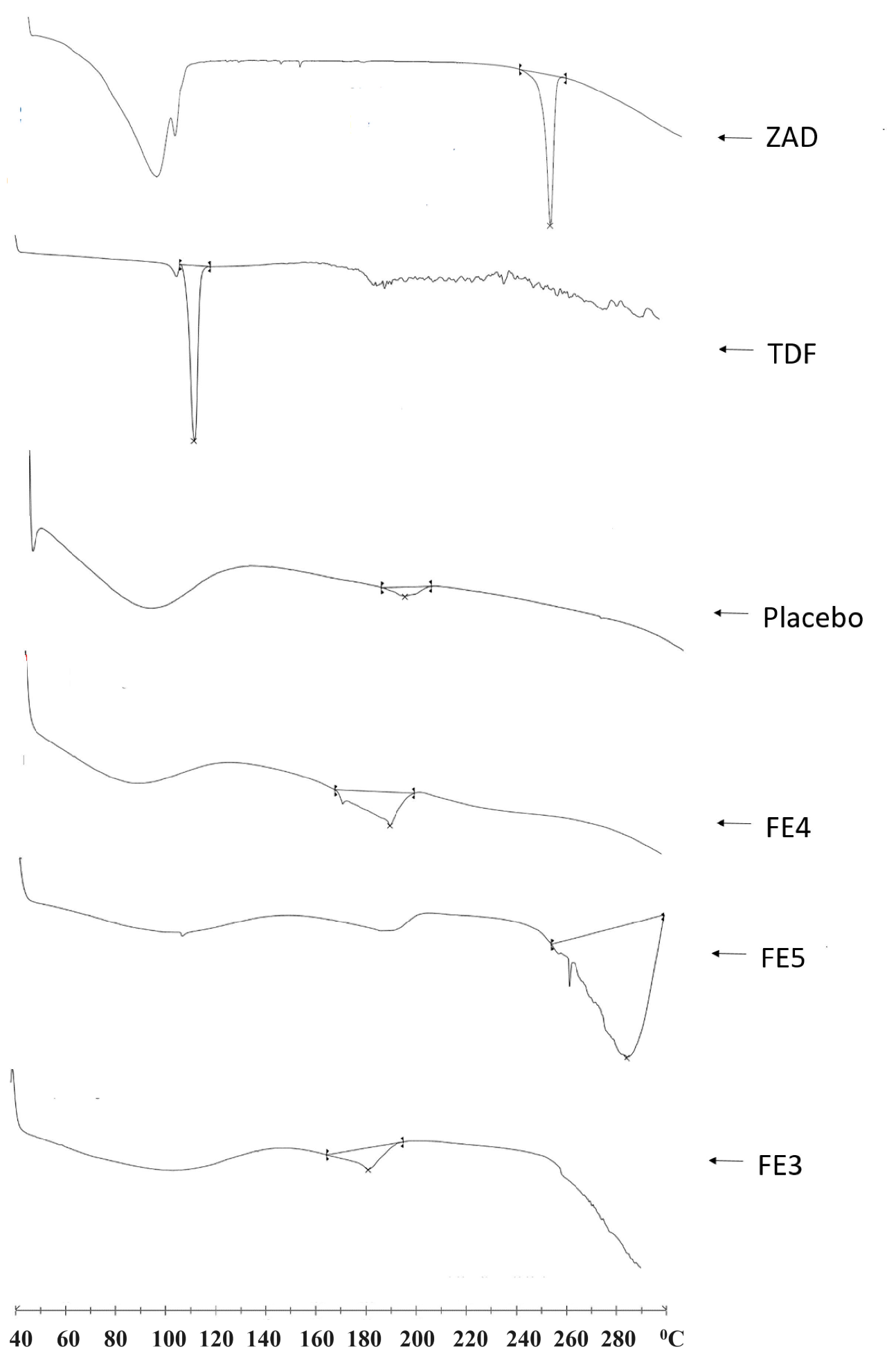


**Figure S2:** DSC thermograms of TDF, ZAD, formulations FE3, FE4, FE5, and placebo film


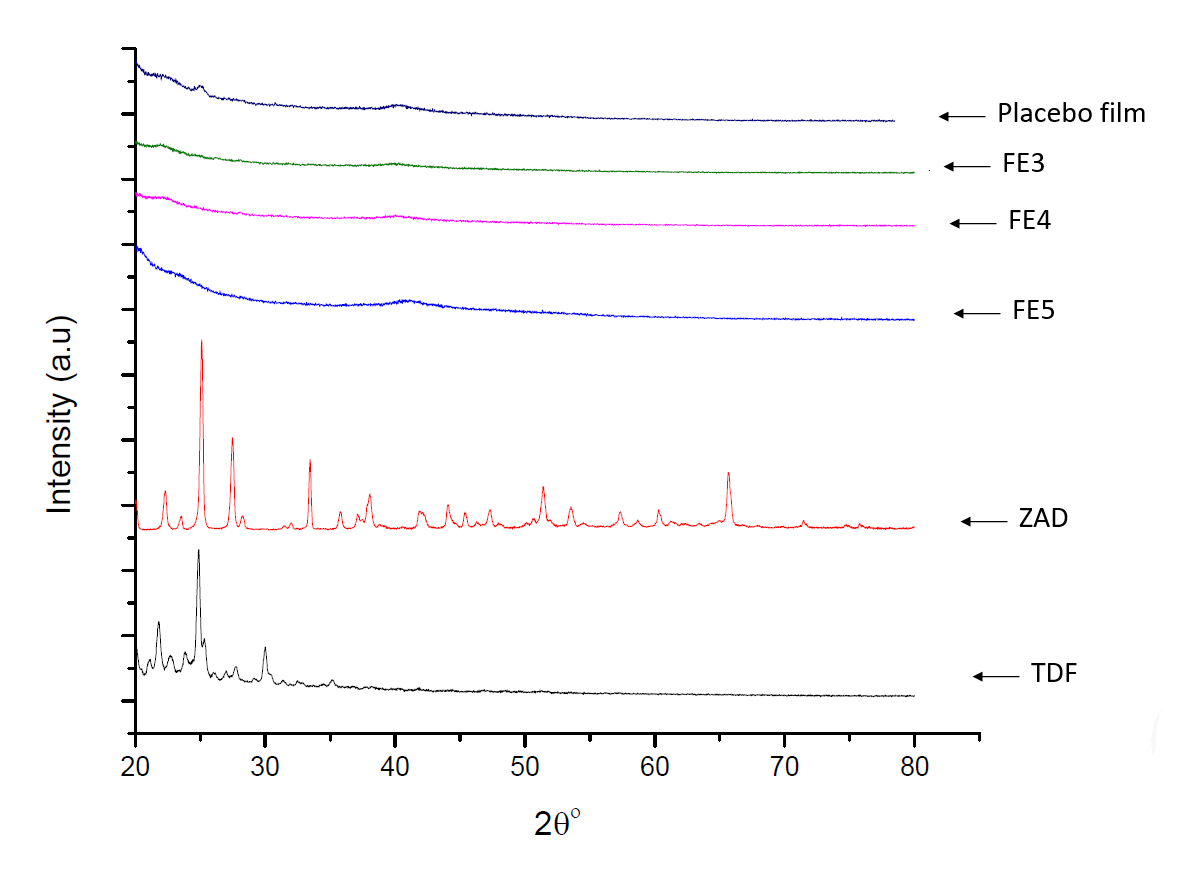


**Figure S3:** PXRD studies of TDF, ZAD, formulations FE3, FE4, FE5, and placebo film


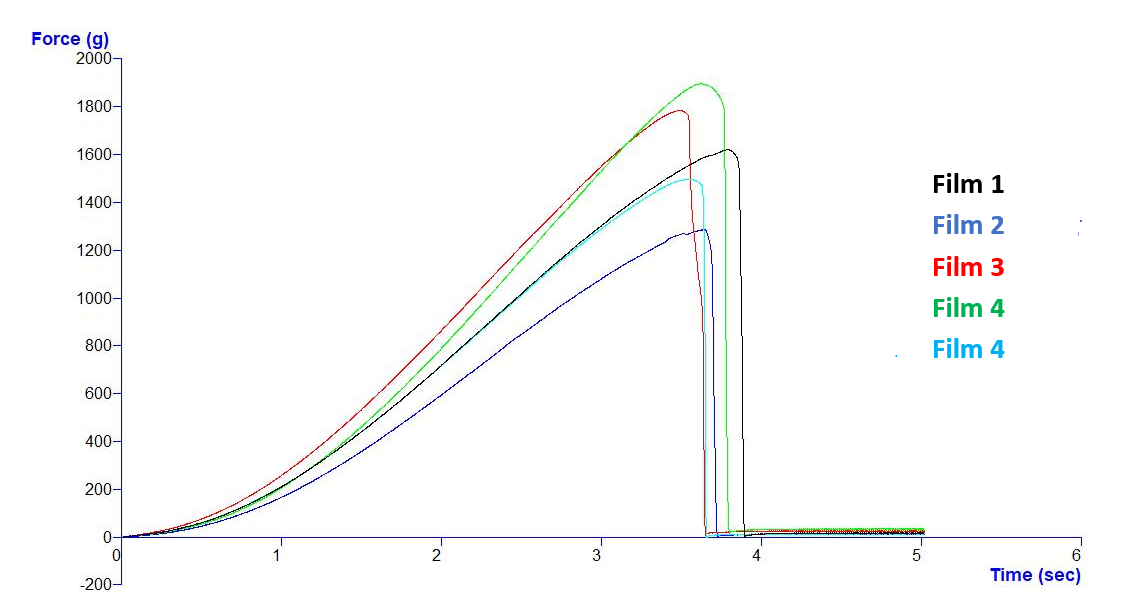


**Fig S4:** Texture analysis of FE3 Film formulation**.**


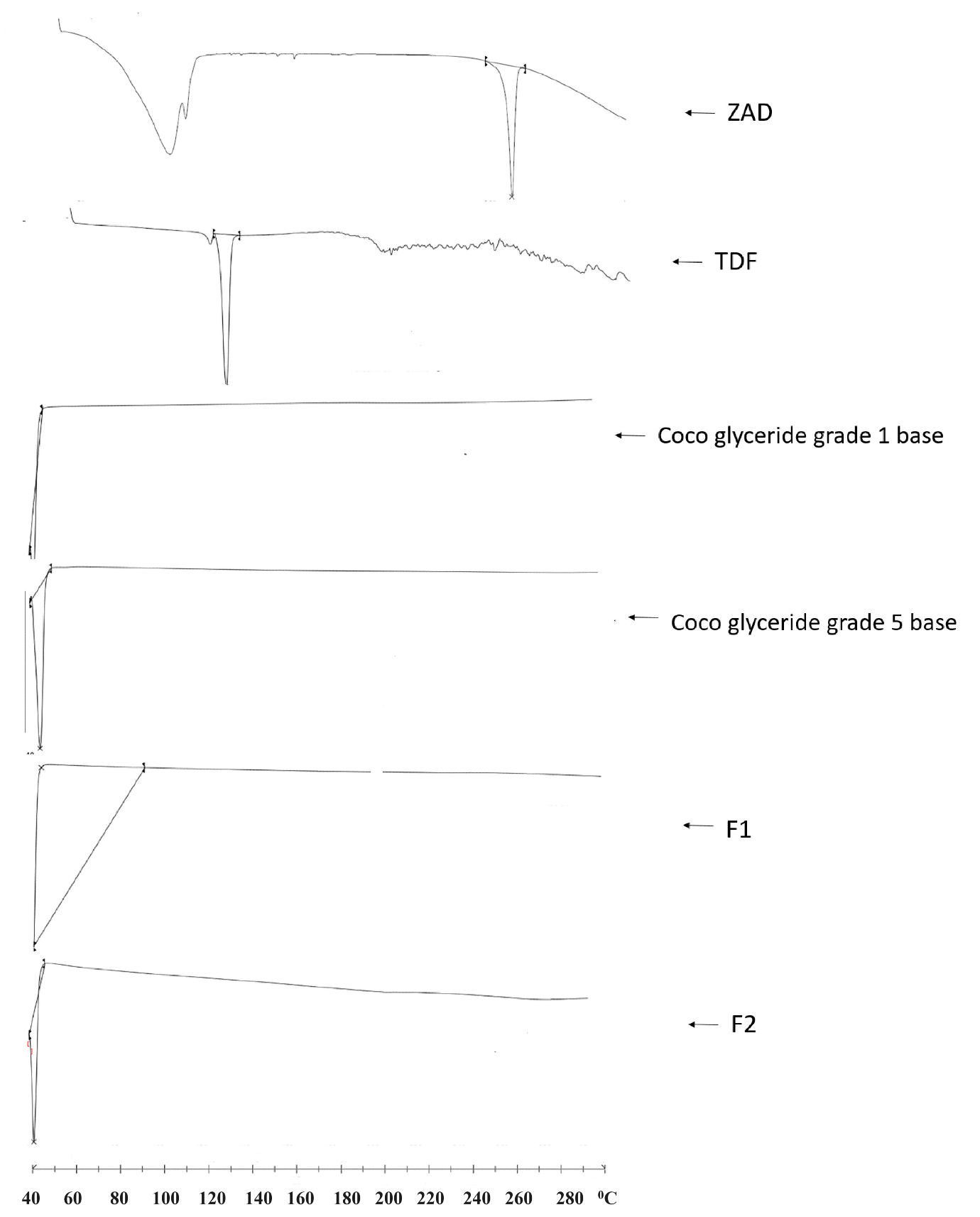


**Figure S5:** DSC thermograms of placebo formulations of Coco glyceride bases grades 1 and 5 and formulations F1, F2


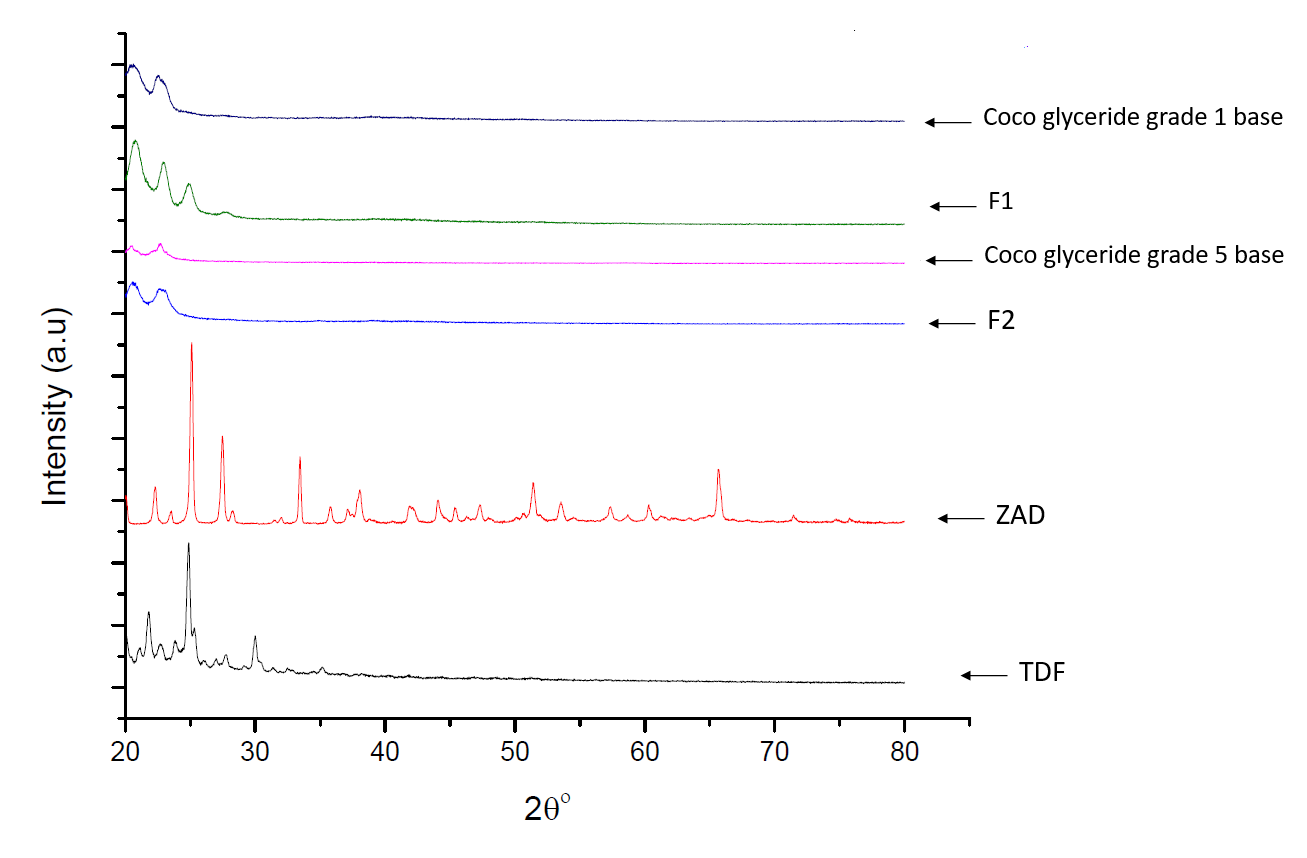


**Figure S6:** PXRD data of TDF, ZAD, placebo pessaries, and formulations F1 and F2


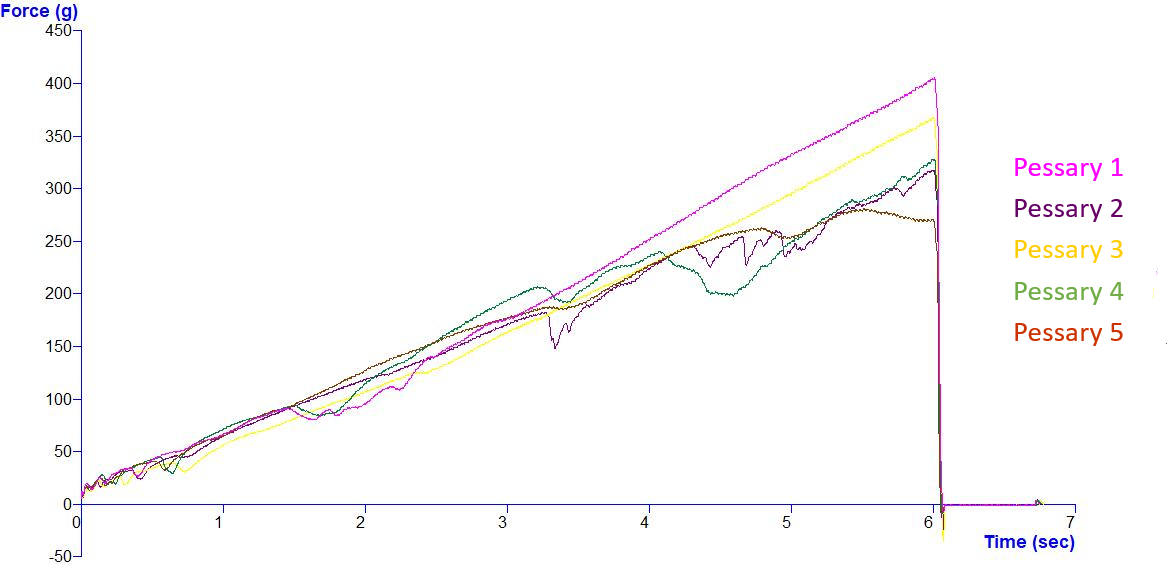


**Figure S7:** Texture analysis of pessary formulation F1.
